# Ethics and biobanks

**DOI:** 10.1038/sj.bjc.6604795

**Published:** 2008-11-25

**Authors:** M G Hansson

**Affiliations:** 1 Department of Public Health and Caring Sciences, Centre for Research Ethics and Bioethics, Uppsala University, Uppsala Science Park, Uppsala SE-751 85, Sweden

**Keywords:** informed consent, privacy, incidental findings, public trust, ethics, biobanks

## Abstract

Biobank research has been the focus of great interest of scholars and regulatory bodies who have addressed different ethical issues. On the basis of a review of the literature it may be concluded that, regarding some major themes in this discussion, a consensus seems to emerge on the international scene after the regular exchange of arguments in scientific journals. Broad or general consent is emerging as the generally preferred solution for biobank studies and straightforward instructions for coding will optimise privacy while facilitating research that may result in new methods for the prevention of disease and for medical treatment. The difficult question regarding the return of information to research subjects is the focus of the current research, but a helpful analysis of some of the issues at stake and concrete recommendations have recently been suggested.

The use of human tissue material in combination with information about disease history and life style in biomedical research has attracted a lot of interest by biomedical scientists, philosophers, lawyers, and different regulatory bodies. The scholarly literature as well as the number of ethical guidelines and legal rules intended to govern biobank research has increased rapidly during the last 10 years. Samples collected for the study of a specific disease should be distinguished from large population studies where samples are collected for future use. However, in practice, the difference between collections may not be so great as samples within the first category may later become of interest for other kinds of studies. The major moral concerns are related to (i) the selection of appropriate information and consent procedures for different research protocols, (ii) the protection of confidentiality of those who submit tissue material or personal information while still facilitating important research, and (iii) how to handle research results or incidental findings that is of potential interest to the donors or to their genetic relatives. Regarding these themes different arguments have been proposed and challenged to an extent that it is now possible to see the beginning of a concordance related to some important positions. I will, in this review, concentrate on this emerging trend, but I will start by going through some of the questions in the current debate.

## Does biobank research imply new ethical challenges?

It has been claimed that biobank research gives rise to new ethical challenges, ‘many of which test our traditional legal concepts, governance provisions and bioethical principles’ (Gibbons and Kaye, 2007, p 204). However, research ethics committees have a rather long experience in balancing interests regarding the collection of sensitive information into databases and the use of genetic information and family history in association with genetic epidemiologic research. It seems to be a well-established and approved practice to select different information and consent procedures for different research protocols (Council of Europe, 1997, Commentary 137 to Article 22; Hansson, 1998), and most countries have national medical registries with sufficient protection of confidentiality, sometimes without the need of individual consent, for example national cancer registries. As Chadwick and Cutter (2007, p 225)have noted:

Arguably, since Gregor Mendel's original experiments with the hereditary characteristics of pea plants, through to James Watson and Francis Crick's identification of the double helix of DNA, the biological sciences have been on a trajectory that seems naturally to culminate in the creation of human genetic research databases or biobanks.

However, even if this may be seen as a natural development, ethical review boards have to deal with new kinds of challenges related to biobank studies that has not been thoroughly reflected in existing guidelines; for example, the selection of an appropriate information and consent procedure for future use of biobank samples, questions about coding of samples and medical data, and the question whether research results should be returned or not (c.f. Auray-Blais and Patenaude, 2006).

## In what sense is public trust at stake?

Public trust is a major concern in association with biobank research. Decreased confidence in biobanking practice may have damaging consequences. If individuals start revoking their consents the banks will not be complete, the possibility to draw scientifically valid conclusions will decrease, and the potential for follow-up examinations and medical treatment will not be fulfilled. In practice, the notion of public trust is a complex phenomenon. As Dixon-Woods *et al* (2008) have shown, there are different ‘publics’ whose trust may be at stake. In an interview study with children and families in paediatric oncology, they found what they called a ‘hybrid social world’, where the patients and their families felt themselves to be in alliance with the nurses, doctors, and scientists. They shared a common goal of providing new treatment opportunities for children with cancer. They were grateful for contributions to science made by earlier patients and were positive to donate samples and give access to medical data in order to benefit future patients. As Dixon-Woods *et al* conclude, this sense of public trust within a community of scientists and patients stands in sharp contrast to a common view where scientists and the public stand in opposition to each other, portraying a picture where the public feel ‘disenfranchised from, and disempowered by, the modern machinery of research…’ (Laurie, 2002, p 311).

Patient groups represent an important part of the general public because they have an experience of participating in research and they are directly concerned by providing samples and information. As witnessed in the study by Dixon-Woods *et al*, they have also a strong sense of solidarity with future patients. However, one should not underestimate the role of the general public and the vulnerability of trust at this level. There is a risk that damaged trust in one area related to medical practice and research may affect other areas as well. On example of this is the organ retention controversy in the United Kingdom that led to the UK Human Tissue Act in 2004, a regulation that has been questioned as not entirely appropriate for biobank research (McHale *et al*, 2007).

It has been suggested that special governance frameworks should be created to promote public trust (Caulfield *et al*, 2003; Cambon-Thomsen *et al*, 2007) and the UK Biobank established accordingly a separate ethics and governance council with this end in mind. Public trust is also believed to require different participatory approaches so that donors of tissue material should have control over how their specimens and data are used and for what purposes. Wolf and Lo (2004) have suggested that ‘tiered consent’, where the donor may permit only some use and require renewed consent for other studies, is a preferable instrument to promote participation. However, this approach is problematic for several reasons.

It should first be observed that ethical review boards and regulatory bodies setting up rules for biobank research are themselves subjects to public trust. Patients and healthy donors have interests at the beginning of the research line, for example being assured about the protection of their integrity and providing tissue material and access to personal data for good scientific reasons, but they have also research interests connected to the potential of providing new treatment and new medical products (Hansson, 2005). On account of long lead times in biomedical and pharmaceutical research, they may not reap a benefit from the actual study but a too strict interpretation of the legal principles governing this kind of research, for example regarding the possibility to use earlier collected samples without a renewed consent, may be detrimental to their general research interests. Under the condition that both the initiation of a new biobank and each new research project emanating from this biobank are examined by the ordinary ethics review boards, there seem to be no need of extra independent governance bodies. Their mandate is unclear, with members often elected by parties directly involved in the biobank effort. For the scientists, they create a new bureaucratic level and they cost money that could be used for research.

Moreover, as argued by Campbell (2007, p 242), to safeguard altruism and trust in biobank research, one should refrain from ‘suggesting that individual donors have ongoing rights to exercise control over uses of their donated materials and the resource itself’. This point has been emphasised also by Shickle (2006, p 516): ‘Providing that there is proper disclosure and so on, then the choice for the individual is to participate on the terms offered or not. There is a ‘negative right’ not to be included in the research without consent … There is no ‘positive right’ for a biobank to be run in such a way just because an individual would like it to be so’.

## Moving towards broad consent to biobank sampling

The selection of appropriate information and consent procedures in association with biobank research has been one of the most contested issues. There are advocates both of an explicit and specified consent for research (Greely, 2007) as well as those critical of the ‘fetishisation’ of consent as the ethical arbiter in biobanking (Laurie, 2008). In support of the last view, Knoppers and Chadwick (2005) have identified a new trend in ethics, where solidarity and reciprocity are guiding ethical principles. They suggest that this ‘symbolize not only a move away from autonomy as the ultimate arbiter … but also an appreciation of the need for a participatory approach’ (ibid p 75). This proposal seems to go against a long and well-established tradition in medical research ethics, where ideas about the respect for patients’ autonomous decisions and their right to say no or to withdraw their consent are vital interests. However, respect for autonomy may still be seen as the basic concern regarding which ideas about solidarity and reciprocity could be developed. The conception of autonomy may arguably in itself include the participatory approach that Knoppers and Chadwick seek.

According to a common view, autonomy is a political concept that basically derives from the ancient world where an individual was autonomous when he took charge of his own affairs, protected from external interference. In contrast to this tradition, Kant defined autonomy as a moral concept (Hansson, 2008). Respect for people's autonomy entails, according to Kant, a respect for their capacity to participate in the formulation of the moral principles that every human being would wish to endorse. Making autonomous decisions in accordance with the Kantian tradition thus involves taking into account of the well-being of others through a judgment of how one's own decisions affect other people's ability to act in a morally responsible way and to attain their own goals. Autonomy in the Kantian tradition is inherently social, with the implication that the working out of legal protections for self-determination and privacy in association with biobank research must simultaneously do justice to both the research subject's independence and to this individuals’ dependence on others for fulfilling mutual interests such as new biomedical knowledge and new treatment opportunities.

As a consequence, several information and consent procedures may be available, which all are legitimate. Respect for autonomy does not automatically lead to the requirements of specific consents, as argued by McQuillan *et al* (2003). From the perspective of the Kantian view on moral autonomy where the individual is called upon to take also other individual's interests into consideration (for example, future members of society), it may be sufficient if there is a democratic instrument available that ensures the individual citizen insight into how the biobank is organised and that principles for balancing of interests at the ethical review boards take all relevant interests into account.

Caulfield (2007) has recently argued against general consent. This has for some time been regarded as the ‘American’ position in comparison with the ‘European’ position, which early on moved towards more general information and consent procedures (Elger and Caplan, 2006). Elger and Caplan suggest now that general consent should be the international standard. The European Council has recently in a draft memorandum taken the view that broad consent to future research use is acceptable (Council of Europe, 2006). This view is shared by several commentators who see this as the emerging trend in biobank ethics (Cambon-Thomsen *et al*, 2007; Haga and Beskow, 2008). The move towards broad or general consent is supported both by empirical surveys and by philosophical arguments (Hansson *et al*, 2006; Wendler, 2006).

According to this view, a general or broad consent is also an informed consent. This understanding has been contested by some (Árnason, 2004; Caulfield, 2007; Greely, 2007). Árnason (2004, p 41) argues that

If we are to preserve a meaningful notion of informed consent for participation in research, it should only be used about specified research where the participants are informed about the aims and methods of a particular research proposal. … There is no such thing as ‘general informed consent’. The more general the consent is, the less informed it becomes. It is misleading to use the notion of informed consent for participation in research that is unforeseen and has not been specified in a research protocol.

However, the success of biobank research implies that large repositories of human tissue material are collected together with well-described and managed clinical and personal data. ‘Biobanks’ represent a wide spectrum of such repositories with associated data, from small clinical sample collections to large research infrastructures with hundreds of thousands of samples. They share nevertheless one characteristic feature. A small biobank can be reused for another purpose as the scientific knowledge develops, and large biobank research platforms are intended for unspecified broad research use in the future. The specific nature of the research is unknown and only general descriptions about the goals of these biobanks are possible, for example for biomedical research or research on diagnose groups. A specific consent to a narrowly described research protocol is not possible and there is a need to ask for a broad or general consent covering future research.

Caulfield and Árnason argue that the traditional meaning of informed consent cannot accommodate these broad and future consents. Consent should be based on specific information, otherwise it is not a valid consent. However, as we in our research team have pointed out earlier, this only begs the question: ‘What is appropriate information? If the information covers all aspects relevant for a person's choice, then that person's consent is appropriately informed. If the essential risk and benefit levels are general to a number of studies, then general information on these studies may be sufficient for the donor of the sample to make an informed decision’ (Hansson *et al*, 2006, p 266). The appearance of several large population-based biobanks in several countries indicates that it is possible to inform about the importance of these research platforms with only general purposes describable. It should be observed that broad or general consent does not imply ‘blanket’ consent to all uses. In agreement with legislation in many countries, the consent refers to use in biomedical research, not to other kinds of uses, for example for forensic use, for investigations of parenthood, or for use by immigration authorities. Biological research in the area of medicine may arguably be of use for many purposes outside medicine and the health-care sector. However, these applications need to be handled by other regulatory frameworks.

## Coding to protect privacy

‘Consent or anonymise’ have for long been the accepted ethical position (Laurie, 2008). However, from a scientific point of view, anonymisation is not a viable alternative for the major bulk of research coming up on gene–environment interaction. The potential of a biobank lies in the possibility to link genetic and biologic data to medical and personal information, and to re-contact donors in order to update this information. Anonymisation has even been regarded as disrespectful to participants (Eriksson and Helgesson, 2005). There is a great variation in the terminology used for protecting privacy. This has been suggested as one of the chief obstacles to successful international collaboration in biobank research by Knoppers *et al* (2007). They have argued that in the absence of ‘common … norms, laws and approaches within a properly harmonised international framework, international collaboration will remain an empty platitude’ (p 311). This is a doubtful position considering the great number of already ongoing successful international collaborations using biobank material. As mentioned by Knoppers *et al* in this article, the Public Population Project in Genomics (P^3^G) is an example of an effort to provide an international Charter while respecting a diversity of national frameworks. There are also regulatory principles already in place.

There is a common understanding of laws for personal data protection that, with some exceptions, it is the individual who has the right of disposition of sensitive personal information. This implies that the patient/donor has the sovereign right to decide if and how personal data and tissue material may be used; for example a ‘yes’ to the use of personal data must be respected by an ethical review board and by the data protection authorities. These authorities may in some instances grant permission to do research using sensitive data without consent from the donor. However, the individual has normally the right to grant such use. This implies that it is essential that the information include possible uses of personal data associated with a research project or the collection of human tissue samples, as well as the measures taken to protect the privacy of the individual donors. It may, for example, include information that genetic analyses will be performed and that international collaboration takes place, which implies the transfer of biological material and data across borders. If the research will or may involve commercial partners and interests, for example future patenting, this may also be included in the information. It is then up to the participant to decide if he or she wants to participate on those terms.

To evade the terminological difficulties, the European authority for supervision of pharmaceutical research (EMEA, 2002) has suggested a nomenclature that seems to fit the needs of current research practice and is in line with the ambitions of different harmonising projects to optimise privacy. They recommend that regarding *anonymous* samples, there are no links to the individual donor (although there may be general descriptions such as ‘man, aged 50–55 years, cholesterol level >240 mg per 100 ml’). *Identified* samples are linked to the individual in a way that makes them immediately identifiable. A *simple code* is a direct link to the individual, usually through a random set of numbers or letters, or a bar code. A *double code* implies that to link the sample and the data to the individual, a second code is needed. *Anonymised* samples are those that have been identified earlier or coded but the identification, or the code and the code key have been destroyed, and thus there is no longer any link to the individual.

The International Conference on Harmonisation of Technical Requirements (ICH) has now (1 November 2007) adopted this nomenclature for the Registration of Pharmaceuticals for Human Use (ICH Harmonised Tripartite Guideline, 2007). The ICH brings together the regulatory authorities of Europe, Japan, and the United States. In the European Union, the Committee for Human Medical Products has endorsed the guidelines, which came into operation in May 2008. The guidelines are made available by the US Food and Drug Administration and by the Pharmaceutical and Medical Safety Bureau in Japan. Taking this recent development into consideration, this nomenclature may now be seen as a significant part of an international Charter regarding coding of human biosamples and data for the protection of privacy of donors donating samples for research.

## Return of information and benefit sharing

As observed by Cambon-Thomsen (2004), the individual participant in a biobank study will typically not benefit by participating. The purpose is to provide knowledge that may benefit a specific disease group or the population at large. This is the common feature of the majority of medical research. Even national population biobanks will have the world population as aimed beneficiaries, rather than their own citizens. A feature of several national legislations is that human body and tissue material cannot be owned or sold. Haga and Beskow (2008, p 526) noted that a Florida court in 2003 (Greenberg v. Miami Children's Hospital Research Institute, Inc.) ‘reaffirmed the *Moore* decision by unambiguously stating that individuals do not have ownership rights in specimens donated for research purposes’. Private benefits are then not available for participants, but there may be either research results or incidental findings that may be of interest to them.

Information about research results of a biobank study is made available through publication in scientific journals. Specific information to individual donors is generally not advisable as it implies assuming a responsibility for the clinical significance for an individual based on information about the odds ratio expressing risk only for a study population. Research groups may not be equipped for assuming such a responsibility. Communicating genetic information implies skills in genetic counselling and the information may be of direct concern to genetic relatives who also must be informed. ‘Misinterpretation can cause potential psychological, social, and economic harm – especially before validation of the clinical significance of the findings. This is particularly true if no relevant treatment or prevention modality to combat the investigated risk is yet available’ (Helgesson *et al*, 2007, p 975). If clinically significant findings are expected to emanate from the research, this implies that a close collaboration has to be set up from the start together with clinical departments and wards that can provide counselling and advice about treatment. There may be different policies for handling research results and handling incidental findings in research even if the clinical significance (clinical validity and clinical utility) may be a common denominator for these policies. Recent recommendations regarding how incidental findings should be handled provides a good start for this policymaking.

In a report from a symposium on the management of incidental findings, Wolf *et al* (2008) have provided a helpful analysis with some central recommendations. They define an incidental finding (IF) as such: ‘An IF is a finding concerning an individual research participant that has potential health or reproductive importance and is discovered in the course of conducting research but is beyond the aims of the study.’ (p 219) The fundamental idea is that the researcher already in the information to the donor/patient should explain how incidental findings of likely health or reproductive importance will be handled. A model for management of these findings will then include collaboration with relevant clinical expertise from the start and preparation of a disclosure scheme depending on the expected net benefits for the individual participant.

## Conclusion

With broad consent emerging as the generally preferred solution for biobank studies and simple instructions available for coding that will protect the privacy of donors there is a good climate for international collaboration that may make progress in biobank research for the benefit of future patients through prevention and treatment. The ethical review boards will play a key role in balancing interests associated with different research protocols. Regarding incidental findings a helpful start has recently been provided.
